# Monocyte Subsets Coregulate Inflammatory Responses by Integrated Signaling through TNF and IL-6 at the Endothelial Cell Interface

**DOI:** 10.4049/jimmunol.1601281

**Published:** 2017-02-13

**Authors:** Myriam Chimen, Clara M. Yates, Helen M. McGettrick, Lewis S. C. Ward, Matthew J. Harrison, Bonita Apta, Lea H. Dib, Beat A. Imhof, Paul Harrison, Gerard B. Nash, G. Ed Rainger

**Affiliations:** * Institute of Cardiovascular Sciences, College of Medical and Dental Sciences, University of Birmingham, Birmingham B15 2TT, United Kingdom;; † Institute of Inflammation and Ageing, College of Medical and Dental Sciences, University of Birmingham, Birmingham B15 2TT, United Kingdom; and; ‡ Department of Pathology and Immunology, University of Geneva, 1211 Geneva, Switzerland

## Abstract

Two major monocyte subsets, CD14^+^CD16^−^ (classical) and CD14^+/dim^CD16^+^ (nonclassical/intermediate), have been described. Each has different functions ascribed in its interactions with vascular endothelial cells (EC), including migration and promoting inflammation. Although monocyte subpopulations have been studied in isolated systems, their influence on EC and on the course of inflammation has been ignored. In this study, using unstimulated or cytokine-activated EC, we observed significant differences in the recruitment, migration, and reverse migration of human monocyte subsets. Associated with this, and based on their patterns of cytokine secretion, there was a difference in their capacity to activate EC and support the secondary recruitment of flowing neutrophils. High levels of TNF were detected in cocultures with nonclassical/intermediate monocytes, the blockade of which significantly reduced neutrophil recruitment. In contrast, classical monocytes secreted high levels of IL-6, the blockade of which resulted in increased neutrophil recruitment. When cocultures contained both monocyte subsets, or when conditioned supernatant from classical monocytes cocultures (IL-6^hi^) was added to nonclassical/intermediate monocyte cocultures (TNF^hi^), the activating effects of TNF were dramatically reduced, implying that when present, the anti-inflammatory activities of IL-6 were dominant over the proinflammatory activities of TNF. These changes in neutrophil recruitment could be explained by regulation of E-selectin on the cocultured EC. This study suggests that recruited human monocyte subsets trigger a regulatory pathway of cytokine-mediated signaling at the EC interface, and we propose that this is a mechanism for limiting the phlogistic activity of newly recruited monocytes.

## Introduction

Peripheral blood monocytes play an important role in the innate immune response against invading pathogens. However, monocytes and cells that differentiate from them in inflamed tissues are also involved in the pathogenesis of a number of diseases (1). A classic example of their contribution to pathogenesis is seen in atherosclerosis, where monocytes recruited to the arterial intima differentiate into proinflammatory “foam cells” upon assimilating cholesteryl ester into storage vesicles (2, 3). Other studies highlight the major role of monocyte-derived macrophages in the pathogenesis of chronic inflammatory diseases. In the autoimmune disease rheumatoid arthritis the frequency of monocytes is increased in the blood and in the diseased synovium of the joint, and there is good evidence that these may contribute to joint destruction (4, 5). The contributions of these cells to pathogenesis occur after the recruitment of monocytes and their differentiation into macrophages; however, there is substantial evidence demonstrating that newly recruited monocytes possess strong phlogistic capabilities and can stimulate endothelial cells (EC), thereby promoting the secondary recruitment of inflammatory leukocytes from the blood (6, 7). Indeed, it appears that in patients with peripheral vascular disease, circulating blood monocytes are more aggressively proinflammatory in such assays, when compared with the monocytes of age-matched heathy control individuals (8).

Interpretation of the above studies is however problematic, as they do not consider the differential functions of the monocyte subpopulations that can readily be demonstrated within the blood. Monocytes are heterogeneous in terms of size and granularity, but specific populations are usually defined by the differential expression of cell surface markers. Human monocytes can be divided into three main subsets based on the expression of CD14 (the LPS coreceptor) and CD16 (FCγRIII): CD14^+^CD16^−^ are often termed classical monocytes; CD14^+^CD16^+^ are intermediate monocytes; and CD14^dim^CD16^++^ are nonclassical monocytes (9). These subsets represent 80–95, 2–11, and 2–8% of the circulating pool, respectively (1, 10–12). Although the rarity of CD16^+^ subsets, in particular nonclassical cells, makes them extremely difficult to analyze functionally, there is evidence that monocyte subsets do possess distinct functional attributes (1, 12). For example, CD16^−^ and CD16^+^ monocytes are transcriptionally distinct, with >250 genes being differentially expressed (13, 14). This leads to marked differences in functions such as phagocytosis and Ag presentation, and it influences the direction of polarization during differentiation into macrophages or dendritic cells (13, 15–17). In the context of inflammation, simple assays measuring the production of cytokines from isolated monocytes show differences in the production of TNF, IL-6, IL-1β, and IL-10, depending upon the subset analyzed (2, 18). The differential expression of chemokine receptors may also influence the migration responses of monocyte subsets. Classical monocytes express high levels of CCR2 and migrate more efficiently to CCL2 in vitro than do other monocyte subsets (19). Additionally, the fractalkine receptor, CX_3_CR1, is highly expressed on intermediate monocytes and has been reported to preferentially support the migration of this subset across EC overexpressing superphysiological levels of CX_3_CL1 (19).

In the context of vascular disease, functionally distinct monocyte populations are of importance, as the proportional representation of different subsets in the blood is known to vary in a disease-specific manner (1, 12). However, studies using monocyte subsets in integrated models of vascular inflammation, where they promote EC activation and support the secondary recruitment of leukocytes, have not been reported. To our knowledge, in this study we provide the first such analysis. In our studies, we have compared classical to nonclassical/intermediate monocytes grouped together. This is because the low numbers of isolated intermediate and nonclassical monocytes did not allow appropriate functional testing of these subsets individually in our assays. In this study, we describe patterns of recruitment and inflammatory function that are dependent on the monocyte subset being assayed, and we also identify a previously unsuspected process of cytokine-mediated regulation of EC activation by monocytes subsets, which moderates the levels of secondary leukocyte recruitment on the endothelium.

## Materials and Methods

### Isolation and culture of human monocytes and monocyte subsets

**FIGURE 1.**
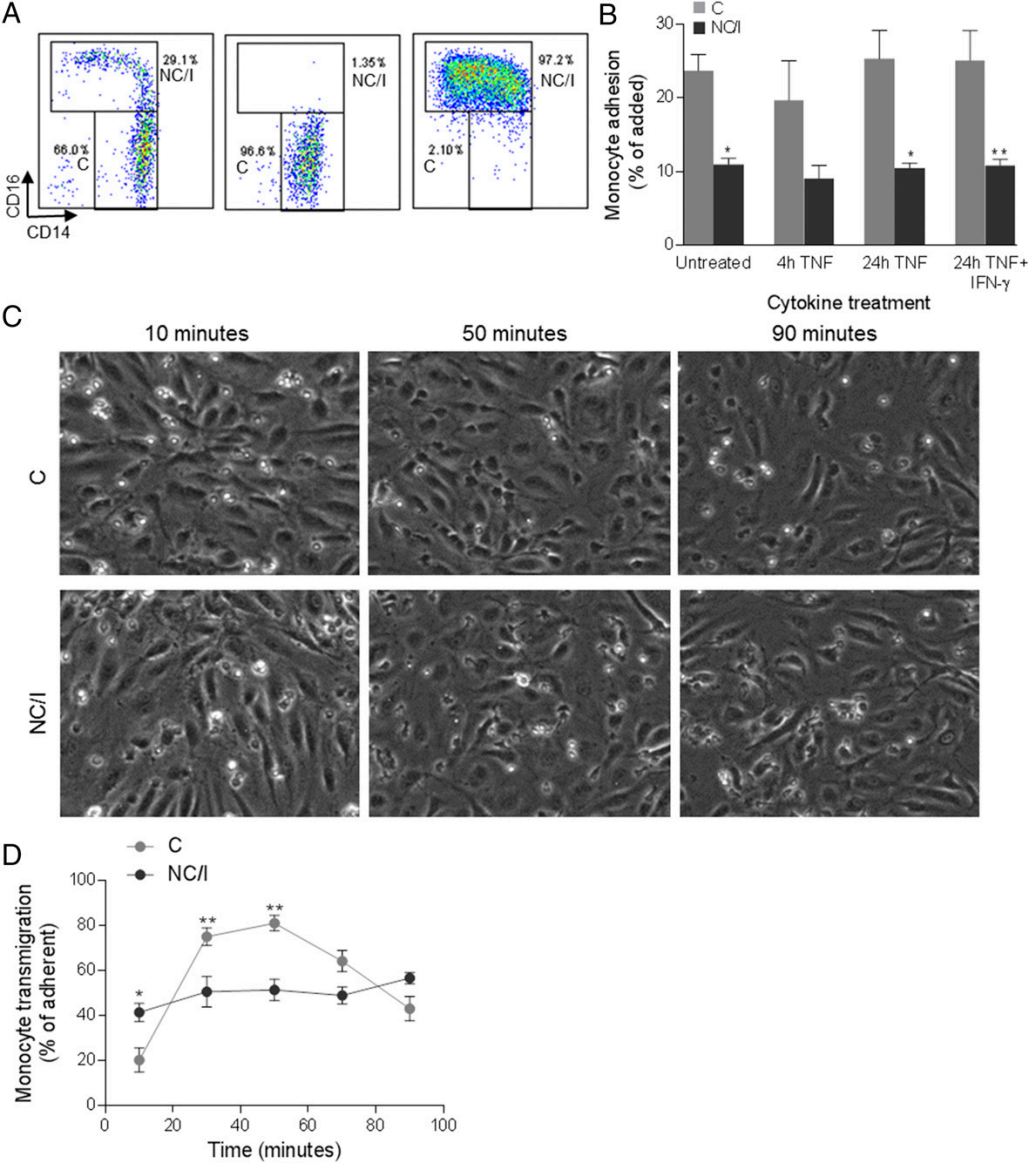
Comparative adhesion and migration of classical and nonclassical/intermediate monocytes on EC. (**A**) Levels of expression of CD14 and CD16 on circulating monocytes and subsets isolated from PBMC measured by flow cytometry (plots representative of >10 preparations). (**B**) Total adhesion of monocyte subsets on unstimulated EC or EC treated with cytokines (*n* = 3–4). (**C**) Representative images of monocyte subsets adhered and transmigrated across unstimulated and TNF stimulated EC. Original magnification ×100. (**D**) Time course of transmigration of monocytes across TNF-stimulated EC (*n* = 3). Data are mean ± SEM. *p ≤ 0.05, **p ≤ 0.01 using a two-ANOVA and Bonferroni posttest (B) and comparison between classical and nonclassical/intermediate monocytes using a two-way repeated measures ANOVA and Bonferroni posttest (D). C, classical monocyte; NC/I, nonclassical/intermediate monocyte.

Blood samples were obtained in EDTA from healthy donors with written informed consent and approval from the University of Birmingham Local Ethical Review Committee (ERN_12-0079C). PBMC and polymorphonuclear neutrophils (PMN) were isolated using Histopaque 1077 and 1119 density media (Sigma-Aldrich, Poole, U.K.) gradient separation (20). Unfractionated or mixed monocytes were isolated from PBMC by positive selection using CD14 microbeads and MACS separation columns (Miltenyi Biotec, Surrey, U.K.). For isolation of classical monocytes, PBMC were depleted of CD16^+^ cells using CD16 microbeads and a MACS separation column (both Miltenyi Biotec) (20). Depleted cells were then enriched for CD14^+^ monocytes using CD14 microbeads (Miltenyi Biotec). Nonclassical/intermediate monocytes were isolated from PBMC using a CD16^+^ monocyte isolation kit (Miltenyi Biotec) including incubation with a nonmonocyte depletion mixture that removes CD15^+^ and CD56^+^ cells in the presence of FCγR blocking Ab. Following this, monocytes were enriched for CD16^+^ monocytes using CD16 microbeads. Purity for both subsets was measured and was routinely ≈95% for both subsets. Purified cells were cultured in M199 (Life Technologies, Paisley, U.K.) containing 10 ng/ml epidermal growth factor (Sigma-Aldrich) and 10% autologous human serum. Monocytes were characterized by surface expression of CD14 and CD16 (Fig. 1A).

### EC isolation and culture

Human umbilical cords were obtained from the Human Biomaterials Resource Centre (University of Birmingham; ethics 09/H1010/75), who collected fully consented tissue from the Birmingham Women’s Hospital National Health Service Trust. Human umbilical vein EC (HUVEC) were isolated from umbilical cords as previously described (21) and cultured in M199 supplemented with 20% FCS, 10 ng/ml epidermal growth factor, 35 μg/ml gentamicin, 1 μg/ml hydrocortisone (all from Sigma-Aldrich), and 2.5 μg/ml amphotericin B (Life Technologies Invitrogen Compounds). HUVEC were grown to confluence in 25-cm^2^ culture flasks (BD Falcon, Oxford, U.K.) precoated with 1% gelatin solution (Sigma-Aldrich). For the monocyte static adhesion and transmigration time-course experiments, primary blood microvascular EC were purchased from PromoCell, cultured in the manufacturer’s recommended medium (EC growth medium; PromoCell), and passaged up to four times prior to use in experiments (20).

### Adhesion assay under static conditions

Confluent primary HUVEC and blood microvascular EC were dissociated using trypsin/EDTA (Sigma-Aldrich) and seeded on 24-well tissue culture plates (Falcon; Becton Dickinson Labware). Seeding density was chosen to yield confluent monolayers within 24 h. TNF (100 U/ml; Sigma-Aldrich) and IFN-γ (10 ng/ml; PeproTech, London, U.K.) were added to confluent monolayers for 4 or 24 h before adhesion assay with monocytes.

Prior to addition of monocytes, EC were washed twice with M199/0.15% BSA to remove residual cytokine. Culture dishes were placed on the stage of a phase-contrast video microscope in a 37°C enclosure. Isolated monocytes (1 × 10^5^ in 300 μl) were added to EC for 7 min and allowed to adhere. Nonadherent cells were gently washed three times with M199/BSA to remove nonadherent cells, and 8–10 fields of view were acquired at 10 min and up to 90 min after adhesion using digital phase-contrast microscopy. Images were analyzed offline using ImagePro software (DataCell, Finchampstead, U.K.). Monocytes in each field were counted. Average cell counts were converted to counts per square millimeters multiplied by the area of the well, divided by the total number of monocytes added, and then multiplied by 100 to express monocyte adhesion as a percentage of the cells added. Adherent monocytes were divided into two populations: (1) phase bright cells on the surface of the EC, and (2) phase dark cells, which had transmigrated across the EC monolayer (22, 23).

### Stimulation of EC by monocyte coculture and PMN adhesion assay in flow conditions

Mixed monocytes or purified classical and nonclassical/intermediate monocytes were seeded onto the inside of porous polyethylene terephthalate culture plastic inserts (Beckton Dickinson), which had been treated with 4% BSA to ensure cell adhesion. The membranes on the inserts had an effective culture area of 0.3 cm^2^, a pore size of 0.4 μm, and a pore density of 1 × 10^8^/cm^2^. Cells were cultured at a density of 0.5 × 10^5^ cells per insert in 24-well plates (Becton Dickinson). Monocytes were allowed to settle on the filters for 45 min, following which confluent HUVEC were seeded (0.5 × 10^5^ EC) onto the apical side of the filter precoated with 1% gelatin. Cells were cocultured together for 24 h. In some experiments, rIL-6 (1 ng/ml; PeproTech), a neutralizing Ab against either IL-6 (10 μg/ml; clone 6708) or a function-blocking Ab against TNF (10 μg/ml; clone 1852; all from R&D Systems), was added when cocultures were established and were present throughout the coculture. In some experiments, supernatants of previous cocultures of classical monocytes with EC were added to fresh cocultures of nonclassical/intermediate monocytes with EC and vice versa.

Viability of isolated monocyte subsets was assessed using calcein-AM (5 μg/ml; Cambridge Bioscience, Cambridge, U.K.) and trypan blue (0.2%; Sigma-Aldrich). We found that all adhered monocytes were calcein positive, and no trypan blue–stained monocytes were detected for either subset in culture after 45 min or coculture after 24 h. Moreover, the number of adherent cells of either subset was not significantly reduced during the 24-h coculture period (Supplemental Fig. 1).

Inserts were incorporated into a parallel plate flow-based adhesion assay as previously described (18, 24). HUVEC on the apical side of the filter formed the base of the channel over which PBS containing 0.15% BSA (PBSA) or isolated, calcein-AM (5 μg/ml; Cambridge Bioscience)–labeled PMN were perfused at a concentration of 1 × 10^6^/ml at a wall shear stress of 0.1 Pa (1 dyne/cm^2^) for 4 min. Following a 2-min wash with PBSA, cells were illuminated with a blue laser (488 nm) and five random fields were acquired using fluorescence microscopy (Andor Technology, Belfast, U.K.), and total leukocyte recruitment (per mm^2^/10^6^ cells perfused) was analyzed and calculated off-line using ImagePro Plus software.

### Analysis of secreted cytokines and chemokines

Luminex kits were purchased from Merck Millipore (Billerica, MA), and the experiment was performed on cell culture supernatants according to the manufacturer’s instructions. Data were collected and analyzed using a Luminex 100 machine (Luminex, Austin, TX).

### Analysis of protein expression by flow cytometry

Isolated monocytes were incubated with Abs against CD14 (1:100 CD14–eFluor 450, clone 61D3; eBioscience) or CD16 (1:100 CD16-PE-Cy7, clone eBioCB16; eBioscience) or appropriate isotype controls for 30 min at 4°C. Plated HUVEC were labeled with Abs against E-selectin (1:100 PE conjugated, clone 68-5H11), VCAM-1 (1:100 FITC conjugated, clone 51-10C9), or ICAM-1 (1:200 allophycocyanin conjugated, clone HA58; all from BD Biosciences) for 30 min at 4°C. Cells were collected using Accutase (Sigma-Aldrich) for 2 min at 37°C and wells were washed with M199 containing 10% FCS. Cells were then washed in 0.15% PBSA and fixed for 5 min in 2% paraformaldehyde. Surface expression was analyzed by flow cytometry on a Dako CyAn (Beckman Coulter, High Wycombe, U.K.), and data were analyzed using Summit software (Dako).

### Analysis of gene expression by quantitative PCR

Total mRNA was extracted using the RNAeasy mini kit (Qiagen, Crawley, U.K.) according to the manufacturer’s protocol. Briefly, EC were collected from the Transwell coculture filters using Accutase, lysed, and then added to a column. After three washes, mRNA was eluted from the column with water. mRNA concentration was measured using a NanoDrop spectrofluorometer (LabTech) and mRNA was stored at −80°C. To convert mRNA to cDNA, random primers (Promega) were annealed to 1 μg of mRNA for 5 min at 70°C, after which the following mastermix was added to give a final volume of 30 μl: 10 U of SuperScript II reverse transcriptase, 10 U of RNaseOUT, 1× SuperScript buffer (all from Invitrogen) and 10 mM dNTPs (Promega). The reaction was run at 37°C for 1 h, followed by 5 min at 95°C. To analyze mRNA, FAM-labeled E-selectin and suppressor of cytokine signaling (SOCS)3 primers and VIC-labeled 18S primers were bought as Assays-on-Demand kits from Applied Biosystems (Warrington, U.K.). Samples were amplified in duplicates using the 7500HT real-time PCR machine (Applied Biosystems) and analyzed using the software package SDS 2.2 (Applied Biosystems). Data were expressed as relative expression units relative to 18S or as fold change (2^−ΔΔCt^ method).

### Statistical analysis

Differences were analyzed using GraphPad Prism software (GraphPad Software, La Jolla, CA) by a paired or unpaired *t* test to analyze differences between classical and nonclassical/intermediate monocytes. One- or two-way ANOVA followed by a Bonferroni post hoc analysis for multiple group comparison was used to compare the different conditions within a monocyte subset. Normality was checked using the Kolmogorov–Smirnov test. A *p* value ≤0.05 was considered significant.

## Results

### The behavior of monocytes upon recruitment is subset-dependent

Migration of monocytes into tissue occurs under both steady-state and inflammatory situations, and this has largely been studied in vivo using mouse models (25–31). In experiments using human monocytes there are limited data indicating differences in the migration of subsets on unstimulated EC monolayers (28). However, the specific patterns of adhesion, transmigration, and reverse transmigration of the subsets in inflammatory conditions remains unclear. In this study we compared the behavior of classical and nonclassical/intermediate monocytes subsets during 90 min on unstimulated EC or monolayers subjected to different regimens of inflammatory activation (i.e., 4 h TNF, 24 h TNF, 24 h TNF plus IFN-γ). Under all conditions, classical monocytes were significantly more efficient at adhering to EC than were nonclassical/intermediate monocytes (Fig. 1B).

Upon analyzing the efficiency of migration we found quite distinct patterns that were dependent on monocyte subset. After 10 min of adhesion, ∼40% of nonclassical/intermediate monocytes had undergone transendothelial migration, and this did not significantly increase during the ensuing 90 min, with the levels of transmigrated nonclassical/intermediate monocytes remaining constant throughout the experiment (Fig. 1C, 1D). In contrast, significantly fewer classical monocytes had undergone transendothelial migration after 10 min (≈20%). However, the number of migrated cells continued to rise to a maximum of 80% at 50 min, which was significantly greater than the efficiency of migration of nonclassical/intermediate monocytes (Fig. 1C, 1D). Most interestingly, we observed that although migrated nonclassical/intermediate monocytes remained under the EC monolayer for the duration of the experiment, classical monocytes underwent reverse transmigration, so that by 90 min ≈50% of the transmigrated population had moved back to the apical surface of the EC monolayer (Fig. 1C, 1D).

### Differences in TNF secretion regulate the levels of EC activation by monocyte subsets

We previously demonstrated that unfractionated monocytes stimulate secondary leukocyte recruitment on EC (6, 7). Using a Transwell coculture model, we investigated the propensity of classical and nonclassical/intermediate monocyte subsets to induce neutrophil adhesion on cocultured EC.

**FIGURE 2.**
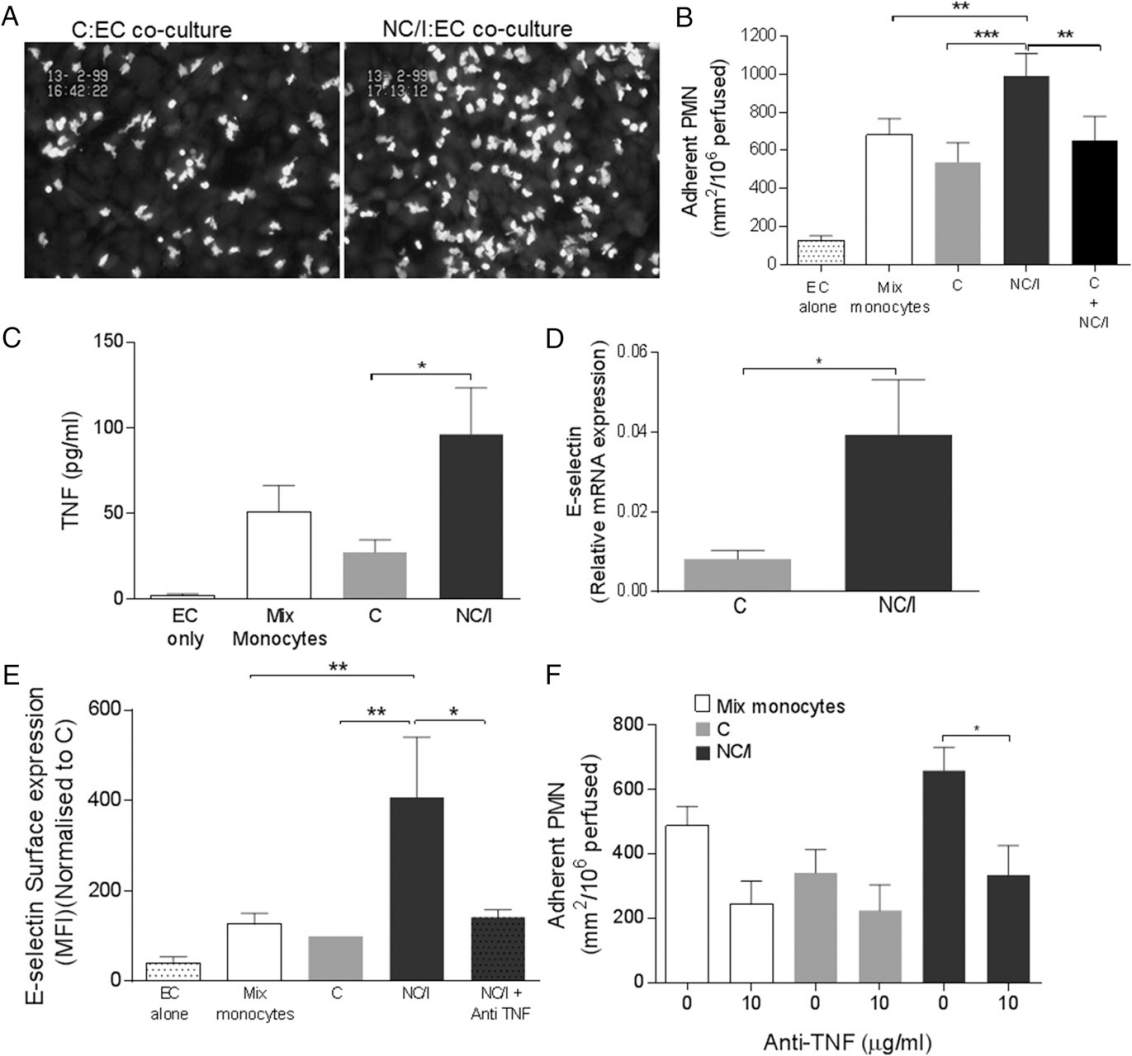
Comparative abilities of classical and nonclassical/intermediate monocyte subsets to activate EC: role of TNF. (**A**) Representative images of neutrophils adhered from flow on EC in coculture with monocyte subsets for 24 h (*n* = 3). Original magnification ×200. (**B**) Recruitment of neutrophils from flow on EC in coculture with monocyte subsets (Mix monocytes indicates unfractionated monocytes; classical plus nonclassical/intermediate monocytes indicates 90% classical plus 10% nonclassical/intermediate monocytes) for 24 h (*n* = 3). (**C**) Concentration of TNF-α (picograms per milliliter) in supernatants of monocyte subsets in coculture with EC for 24 h measured using a Luminex assay (*n* = 3). (**D**) mRNA expression of E-selectin on EC alone or in coculture with monocyte subsets for 24 h (*n* = 7–9). (**E**) Surface expression of E-selectin on EC following coculture with monocyte subsets for 24 h, measured by flow cytometry (*n* = 4–5). TNF was neutralized using an Ab at 10 μg/ml during the 24-h coculture with nonclassical/intermediate monocytes. Data were normalized to expression of E-selectin on EC alone. (**F**) Recruitment of neutrophils from flow on EC cocultured with monocyte subsets for 24 h, with or without 10 μg/ml anti-TNF Ab (*n* = 3). Data are mean ± SEM from *n* experiments. *p ≤ 0.05, **p ≤ 0.01, ***p ≤ 0.001 comparison between classical and nonclassical/intermediate monocytes using repeated measures ANOVA and a Bonferroni posttest (A), ANOVA and a Bonferroni posttest (B, C, and E), an unpaired *t* test (D), and a two-way ANOVA and a Bonferroni posttest (F). C, classical monocyte; NC/I, nonclassical/intermediate monocyte.

In the absence of exogenous cytokines, EC that were cultured on Transwell inserts in the absence of monocytes did not support the efficient adhesion of flowing neutrophils (Fig. 2A, 2B). In contrast, the coculture of EC with unfractionated monocytes for 24 h, on the opposite sides of 0.4-μm-pore Transwell membranes, promoted the adhesion of flowing neutrophils (Fig. 2B), consistent with our previous observations (6, 7). Interestingly, though, when isolated monocyte subsets were cocultured with EC, nonclassical/intermediate monocytes could induce significantly higher levels of neutrophil adhesion than classical monocytes (Fig. 2A, 2B). Indeed, classical cells, which represent ≈90% of the circulating population, stimulated EC to the same extent as unfractionated monocytes. Importantly, we found that the viability of classical and nonclassical/intermediate monocytes was unafected after 24 h of culture, meaning that the differences in EC activation and neutrophil recruitment were not based on different numbers of monocytes in coculture (Supplemental Fig. 1). To demonstrate that the subset isolation procedure had not unduly influenced the inflammatory activity of the monocytes, we mixed the isolated subsets at physiological ratios (90% classical and 10% nonclassical/intermediate) and observed the same profile of neutrophil adhesion as seen in unfractionated monocytes (Fig. 2B).

**FIGURE 3.**
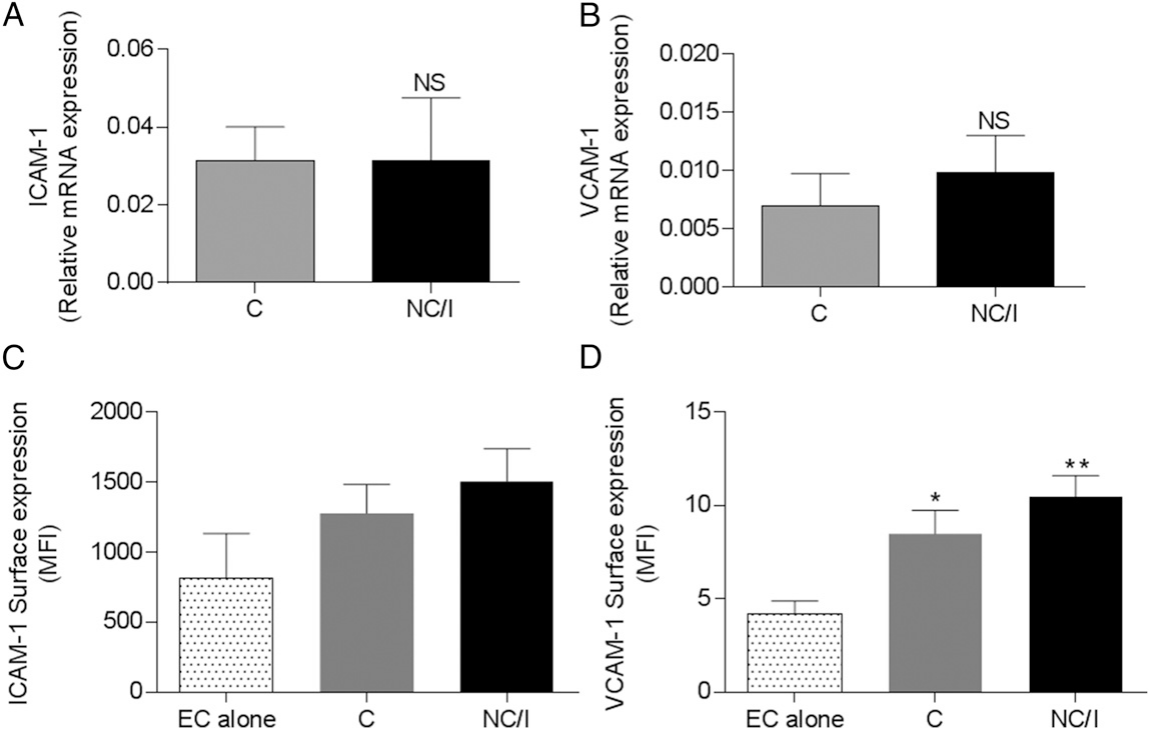
Expression of VCAM-1 and ICAM-1 in EC in coculture with monocyte subsets. (**A** and **B**) mRNA expression of VCAM-1 (A) and ICAM-1 (B) on EC in coculture with monocyte subsets for 24 h (*n* = 7–9). (**C** and **D**) Surface expression of VCAM-1 (C) and ICAM-1 (D) on EC following coculture with monocyte subsets for 24 h, measured by flow cytometry (*n* = 4–5). Data are normalized to classical monocyte mean fluorescence intensity (MFI). Data are mean ± SEM from *n* experiments. *p ≤ 0.05, **p ≤ 0.01, NS, not significant using unpaired *t* test (A and B) and ANOVA and a Dunnet posttest to EC alone (C and D). C, classical monocyte; NC/I, nonclassical/intermediate monocyte.

Secretion of TNF is the mechanistic pathway by which unfractionated monocytes have been shown to activate EC (6, 7). Thus, we tested whether this was also the mechanism by which fractionated monocytes could stimulate cocultured EC. When we assayed for the production of this cytokine in coculture supernatants, we found that EC alone did not generate detectable levels (Fig. 2C). Nonclassical/intermediate monocytes in coculture with EC generated substantial levels of TNF (≈100 pg/ml) that were higher than levels in cocultures with classical monocytes or unfractionated monocytes (Fig. 2C). As neutrophil recruitment from flow in this model is supported by E-selectin (6, 7), we verified that this receptor also showed differential expression in response to EC coculture with the different monocyte subsets. The expression of both E-selectin mRNA and protein was higher in nonclassical/intermediate monocyte cocultures and reduced when TNF was blocked (Fig. 2D, 2E). The dominant role of this cytokine in promoting neutrophil recruitment was confirmed when a function neutralizing Ab against TNF was added to cocultures. This resulted in significant reductions in the expression of E-selectin and the recruitment of flowing neutrophils to EC cocultured with nonclassical/intermediate monocytes (Fig. 2E, 2F). Although we observed an upregulation of ICAM-1 and VCAM-1 expressions on HUVEC in coculture with both monocyte subsets compared with EC alone, we did not find differences in expression on HUVEC cocultures with classical and nonclassical/intermediate monocytes at either the mRNA (Fig. 3A, 3B) or protein (Fig. 3C, 3D) levels.

### IL-6 generated by classical monocytes regulates EC activation

**FIGURE 4.**
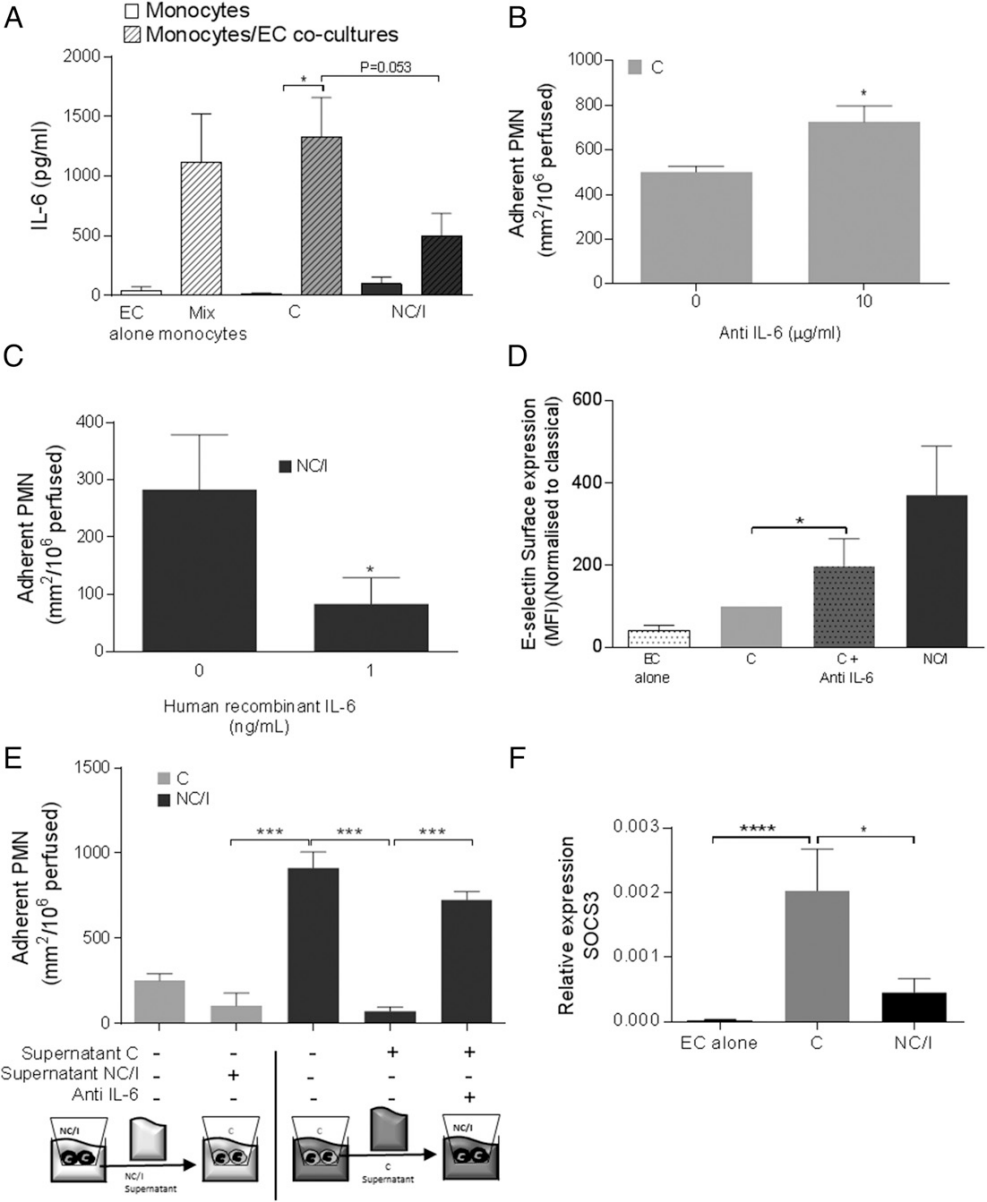
Role of IL-6 in regulating EC activation by monocyte subsets. (**A**) Concentration of IL-6 (picograms per milliliter) in supernatants of monocyte subsets in coculture with EC for 24 h measured using a Luminex assay (*n* = 3). (**B**) Recruitment of neutrophils from flow on EC cocultured with classical monocytes, with or without neutralizing Ab against IL-6 (10 μg/ml) (*n* = 4). (**C**) Recruitment of neutrophils on EC cocultured with nonclassical/intermediate monocytes, with or without rIL-6 (1 ng/ml) (*n* = 4). (**D**) Surface expression of E-selectin on EC following coculture with monocyte subsets for 24 h, measured by flow cytometry. Data were normalized to expression of E-selectin on cocultures with classical monocytes (*n* = 4–5). (**E**) Recruitment of neutrophils from flow on EC cocultured with monocyte subsets for 24 h: effect of adding supernatants from cocultures with either subset, and of neutralizing Ab against IL-6 (10 μg/ml) (*n* = 3). (**F**) mRNA expression of SOCS3 on EC alone or in coculture with monocyte subsets for 24 h (*n* = 7–9). Data are shown as mean ± SEM. *p ≤ 0.05, ***p ≤ 0.001, ****p ≤ 0.0001, comparison between classical and nonclassical/intermediate monocytes using an unpaired *t* test (D and F), a paired *t* test (B and C), and ANOVA and a Bonferroni posttest (A and E). C, classical monocyte; NC/I, nonclassical/intermediate monocyte; PMN, polymorphonuclear neutrophil.

We have previously demonstrated that IL-6 contributed to the reduction in leukocyte adhesion when EC were cultured in direct contact with mesenchymal stem cells or in close proximity to fibroblasts (32, 33). Upon analysis of the profile of cytokine secretion in coculture supernatants, we observed that IL-6 was also differentially released by monocyte subsets cocultured with EC (Fig. 4A). Classical monocyte cocultures produced significantly higher concentrations of IL-6 compared with nonclassical/intermediate monocytes. As we have previously shown that IL-6 can regulate neutrophil trafficking in coculture experiments (32), we neutralized its activity and assayed for the recruitment of flowing neutrophils. Neutralizing IL-6 in cocultures containing classical cells led to a significant increase in the level of neutrophil recruitment compared with untreated cocultures (Fig. 4B), implying that IL-6 was operating as a functional break on endothelial responses to TNF. As IL-6 was operating in an autocrine fashion in cocultures of classical monocytes and EC, we used rIL-6 to determine whether we could regulate neutrophil recruitment in nonclassical/intermediate monocyte–based cocultures in a paracrine fashion. Using a concentration consistent with that produced by classical monocyte cocultures (1 ng/ml), we could dramatically reduce neutrophil recruitment in nonclassical/intermediate monocyte cocultures upon the addition of exogenous IL-6 (Fig. 4C). Importantly, this was predicated on regulation of E-selectin expression, which was dramatically upregulated on EC cocultured with classical monocytes in the presence of IL-6 blockade (Fig. 4D).

Having demonstrated that IL-6 could function to regulate neutrophil trafficking in a paracrine fashion in our model, we tested whether crosstalk between monocyte subsets occurred through their secreted cytokines. Importantly, we observed no other differences in levels of secreted cytokines (such as IL-1β, IL-4, CXCL8, IL-10, CXCL10, CCL2, LTα, Flt3L, CX3CL1, GM-CSF, CXCL1, or CCL3) in the cocultures measured using a multiplexed immunofluorometric assay (Supplemental Fig. 2). Using the conditioned supernatants from monocyte subset–specific cocultures, we conducted a number of supernatant crossover experiments. The addition of matched supernatants to monocyte subset cocultures (i.e., classical to classical or nonclassical/intermediate to nonclassical/intermediate monocytes) did not affect patterns of neutrophil recruitment (Fig. 4E). The addition of nonclassical/intermediate monocyte supernatant (TNF^hi^/IL-6^lo^) to classical monocyte cocultures (TNF^lo^/IL-6^hi^) did not increase neutrophil recruitment (Fig. 4E). Interestingly, the addition of classical (TNF^lo^/IL-6^hi^) coculture supernatants to nonclassical/intermediate monocytes (TNF^hi^/IL-6^lo^) cocultures abolished neutrophil recruitment (Fig. 4E). Importantly, both of these experimental outcomes show that IL-6 is the dominant functional cytokine and downregulates the TNF response when it is present. This was confirmed when high levels of neutrophil adhesion were restored on nonclassical/intermediate monocytes cocultures in the presence of classical coculture supernatant treated with an IL-6 function neutralizing Ab (Fig. 4E).

**FIGURE 5.**
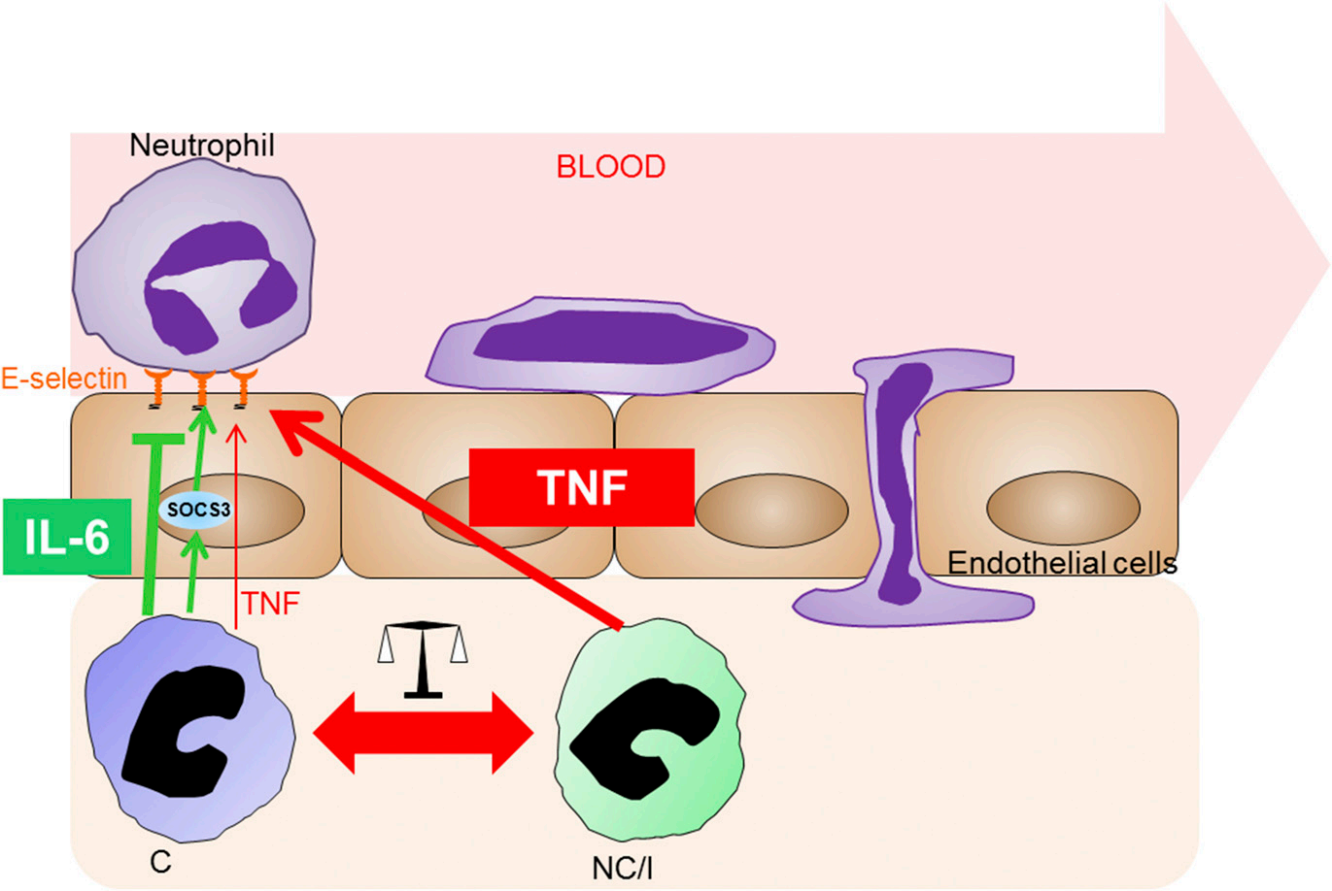
Cytokines secreted by monocyte subsets balance EC activation. Nonclassical/intermediate monocytes generate high levels of TNF once in proximity with EC, which causes upregulation of E-selectin expression at the EC surface. This in turn promotes secondary leukocyte recruitment. In normal inflammatory conditions, classical monocytes regulate the extent of nonclassical/intermediate monocyte–induced EC activation by secreting IL-6, which reduces surface expression of E-selectin via upregulation of SOCS3, a negative regulator of the JAK/STAT signaling pathway. In disease, potential changes in classical and nonclassical/intermediate monocyte recruitment may alter the balanced control of inflammation mediated by the regulation mediated by both subsets. C, classical monocyte; NC/I, nonclassical/intermediate monocyte.

Recent studies have indicated that IL-6–mediated regulation of the transcription factor SOCS3, which is a negative regulator of the JAK/STAT signaling pathway, can downregulate expression of proinflammatory genes in EC, including E-selectin, thereby influencing leukocyte trafficking responses (34). In this study, we found that in the presence of classical monocytes (and thus high IL-6) there was high EC expression of SOCS3 mRNA (Fig. 4F). In contrast, in the presence of nonclassical/intermediate monocytes (low IL-6), EC showed a much lower expression of SOCS3 mRNA (Figs. 4F, 5).

## Discussion

We have previously demonstrated that in coculture, a mixed population of monocytes can activate EC, leading to the recruitment of flowing neutrophils (6, 7). In this study, we extended these observations using monocyte subsets and have identified, to our knowledge for the first time, a differential ability of classical and nonclassical/intermediate monocyte subsets to activate EC (Fig. 5). To our knowledge, we first demonstrated that monocyte subsets behave differently in terms of recruitment, transmigration and reverse transmigration on EC. Indeed, we have shown that more classical monocytes are recruited on both unstimulated and cytokine-stimulated EC, which is in agreement with other published data (28). Although the efficiency of migration of nonclassical/intermediate monocytes was low (≈40%), those cells that did migrate traversed the monolayer rapidly, remaining underneath for the duration of the experiment. In contrast, classical cells continued to migrate for 50 min, leading to an efficiency of migration in >80%. Interestingly, nonclassical/intermediate monocytes did not possess the capacity to reverse transmigrate similar to classical monocytes. The physiological relevance of reverse migration is still unclear; however, this process has now been observed for neutrophils, lymphocytes, and monocytes both in vitro and in vivo (16, 26, 35–37). Thus, it is interesting that the nonclassical/intermediate monocytes do not undertake this behavior. This implies lack of appropriate receptors to respond to signals for reverse migration, or alternatively possession of receptors that respond to retention signals within the subendothelial environment.

As nonclassical/intermediate monocytes are not able to return to the circulation under inflammatory conditions, this raises the issue of their potential capacity to influence EC activation. Our model of monocyte coculture with EC allowed us to investigate processes that may occur in the microcirculation during inflammation or in the artery wall during atherogenesis. In these conditions, monocytes are efficiently recruited (2, 38) and can activate EC to recruit more leukocytes (6, 7). In agreement with published observations on the surface expression of markers and cytokine expression in monocyte subsets (12), we observed a higher capacity of nonclassical/intermediate monocytes to secrete TNF and higher secreted levels of IL-6 in classical monocytes. We show that nonclassical/intermediate monocytes are able to efficiently activate EC to recruit circulating neutrophils because of their capacity to secrete TNF, which upregulates the expression of E-selectin on EC allowing efficient capture of flowing neutrophils. In contrast, classical monocytes are able to secrete IL-6, which downregulates the expression of E-selectin on EC via induction of SOCS3, thereby reducing neutrophil recruitment.

Other studies have shown different expression of surface markers and cytokine profiles on nonclassical and intermediate monocytes as well as different phagocytosis, Ag processing and presentation, proangiogenic and patrolling behaviors, and proportions in diseases (reviewed in Ref. 39). In our studies, we are not able to attribute the effects of nonclassical/intermediate monocytes to a more specific subpopulation due to limited material available. Indeed, isolation of intermediate nonclassical and intermediate monocytes is not achievable using magnetic beads to date. Moreover, the numbers of either subset are limiting when they are further subdivided. Indeed, even using pooled nonclassical/intermediate monocytes, we were at the limit of functionality in our coculture assays when making multiple comparisons. A more detailed analysis would be of great interest, but to conduct these in exacting multicellular systems is going to prove extremely demanding from a technical point of view. Indeed, this probably explains the extremely limited data available on the function of these two subsets.

It is clear that TNF is a proinflammatory mediator able to induce the expression of E-selectin on EC and promote leukocyte recruitment (6). Although IL-6 is often termed an inflammatory cytokine, it does not drive leukocyte recruitment per se (33). Rather, previous studies have shown the ability of IL-6 either alone or in conjunction with TGF-β1 to modify inflammatory infiltrates by inhibiting recruitment (40, 41) or by switching recruitment from neutrophils to mononuclear leukocytes (42–44). In this context, we have reported that the inhibitory actions of IL-6 are also exploited by Kaposi’s sarcoma herpesvirus to reduce neutrophil migration (34). Indeed, we have also shown that IL-6 alone, or in combination with TGF-β1, was responsible for the immunosuppressive effects of glomerular podocytes (45), mesenchymal stem cells (32, 46), and healthy dermal fibroblasts (33). In these in vitro studies, leukocyte adhesion to inflamed endothelium was reduced in the presence of IL-6. In contrast, stromal cells from chronically inflamed sites, such as the rheumatoid joint or atherosclerotic plaque, transform the bioactivity of IL-6 and/or TGF-β1, making them act in a stimulatory (47) or proinflammatory manner (33, 48). Consequently, the mode of action of IL-6 is contextual, defined by other cofactors present within the local milieu (49). Thus, in healthy conditions it is likely that classical monocyte–derived IL-6 controls the magnitude of the inflammatory response by limiting access of other leukocytes, and this may favor resolution of the inflammatory process. Although IL-6 can act in concert with TGF-β1 to mediate its immunosuppressive effects (32, 33, 41, 46), this also appears to be context specific, with IL-6 able to act in an anti-inflammatory manner, independent of TGF-β1 (34, 45, 50). In our model, blockade of TGF-β1 in monocyte subsets in coculture with EC did not alter neutrophil recruitment (data not shown), suggesting that IL-6 is acting through another mechanism of action to regulate TNF signaling. Our work indicates a role for SOCS3 in the IL-6–modulated responses of EC to TNF, in particular regulation of E-selectin on EC. The SOCS family consists of eight structurally related proteins that are induced by different cytokines and other stimuli (51) and attenuate signaling by blocking JAK tyrosine kinase activity or STAT activation (51). In our study, SOCS3 is expressed at low levels in unstimulated EC and is significantly upregulated on EC in coculture with classical monocytes (IL-6^hi^/TNF^lo^). Supporting the idea that SOCS3 can have a role in the modulation of neutrophil recruitment during inflammation, IL-1–induced acute inflammatory arthritis was particularly severe in SOCS3 knockout mice (52), a model characterized by increased numbers of neutrophils in the inflamed synovium. Taken together, these data support the idea that SOCS3 is initially upregulated by IL-6 and in turn suppresses signaling of other mediators, such as TNF and IL-1. Indeed, this idea is supported by a previous study that showed that SOCS3 overexpression in a colon cancer cell line inhibits subsequent TNF-induced p65 phosphorylation (53). This was also shown in EC in which the Kaposi’s sarcoma–associated herpesvirus inhibits TNF induced neutrophil migration by upregulating IL-6 release, which in turns activates SOCS3 (34).

Differential cytokine-mediated regulation of EC activation by the monocyte subsets in an inflammatory environment is a novel observation. We show that supernatants with high levels of IL-6 from classical monocyte cocultures with EC are able to downregulate nonclassical/intermediate monocyte inflammatory responses driven by TNF. In contrast, nonclassical/intermediate monocyte supernatants rich in TNF were not able to counteract the anti-inflammatory properties of IL-6 in classical monocyte cocultures. Thus it seems likely that in the context of physiological resolving inflammation, rapid responses to inflammatory stimuli can be mediated by nonclassical/intermediate monocytes, as they are able to transmigrate rapidly and efficiently across the endothelium. However, subsequent recruitment of classical cells would control the extent of such a response, as these IL-6–secreting cells become represented in the inflammatory infiltrate. In the context of chronic disease where the ratio of the subsets may be skewed toward nonclassical/intermediate monocytes, one might predict a bias to prolonged or exaggerated inflammatory responses that could contribute to the development of disease. Indeed, such a scenario is reported in pathological conditions such as sepsis (54), tuberculosis, metastatic cancer, asthma (55), and HIV infection (17) as well as coronary heart disease (56). Thus, manipulating the abundance and biological activity of monocyte subsets may have utility in moderating pathogenesis in a number of diseases.

## Supplementary Material

Data Supplement
