# Supplementary material for: Monocyte Subsets Coregulate Inflammatory Responses by Integrated Signaling through TNF and IL-6 at the Endothelial Cell Interface
Source: J Immunol. 2017 Feb 13;198(7):2834–43. doi: 10.4049/jimmunol.1601281 (PMC5357784; doi:10.4049/jimmunol.1601281)
Supplement: Data Supplement [file JI_1601281.zip › JI_1601281_Supplemental_Figures_1.pdf]

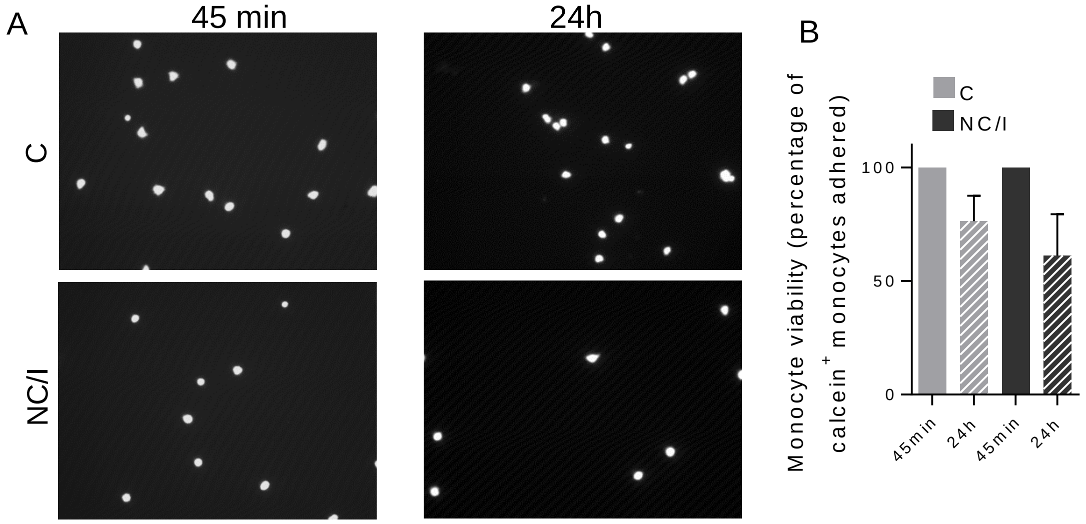

SUPPLEMENTARY FIGURE 1. Viability of isolated monocytes subsets over 24 hours. A, Representative images of calcein positive live monocytes subsets, classical (top) and non-classical/intermediate (bottom) monocytes in culture for 45 minutes and 24 hours. **B,** Percentage of calcein positive isolated monocyte subsets adhered after 45 minutes and 24 hours of culture, n=3. Data are mean ± s.e.m from n experiments. No significant difference between 45 min and 24h by paired t-test. C: classical and NC/I: non-classical/intermediate monocytes.

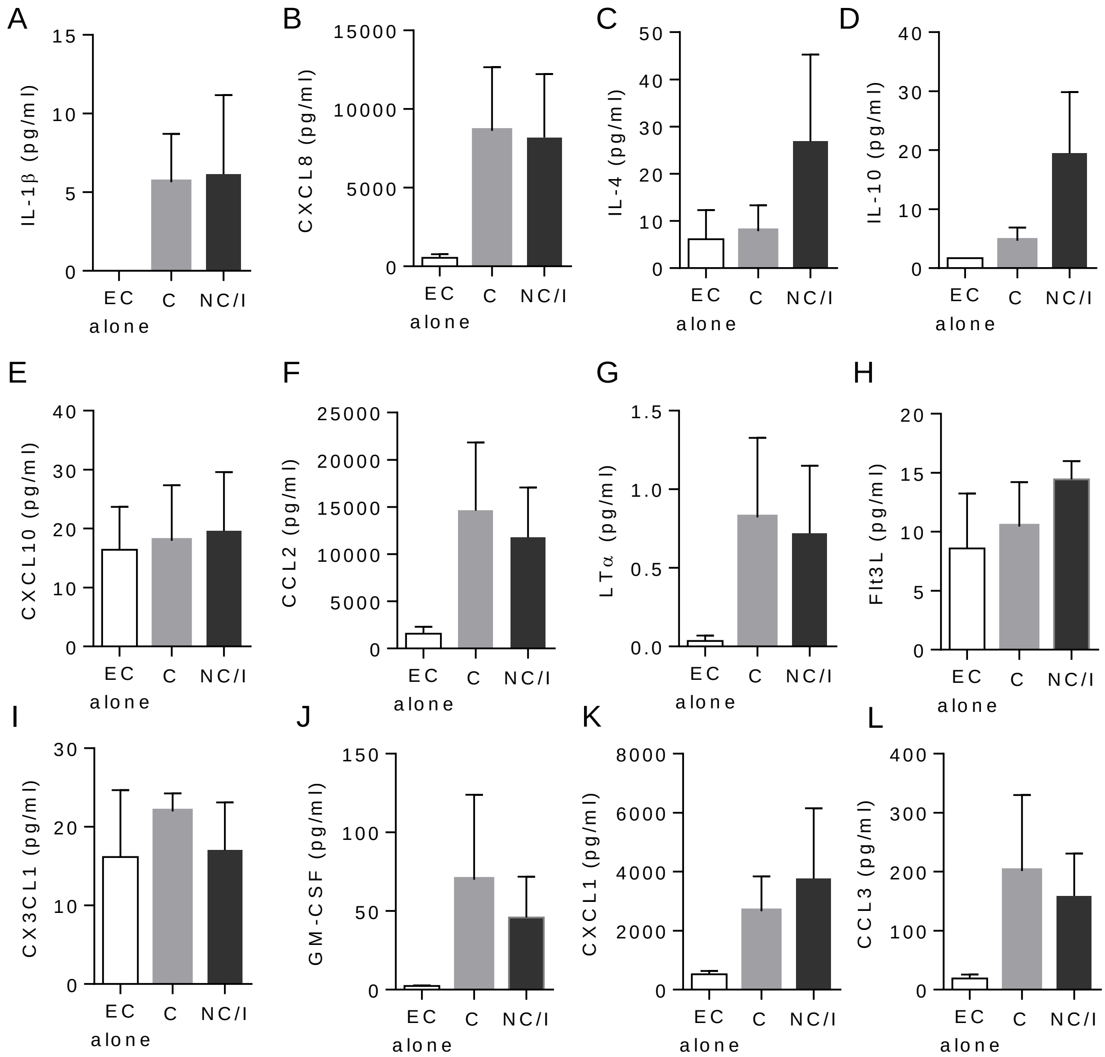

SUPPLEMENTARY FIGURE 2. Cytokines levels in supernatants of monocyte subsets in co-culture with EC. A-L, Concentration of IL-1β (**A**), CXCL8 (**B**), IL-4 (C), IL-10 (**D**), CXCL10 (**E**), CCL2 (**F**), LTα (**G**), Flt3L (**H**), CX3CL1 (**I**), GM-CSF (**J**), CXCL1 (**K**) and CCL3 (**L**) in supernatants from EC in co-cultures with classical and non-classical/intermediate monocytes, n=3-6. Data are mean ± s.e.m from n experiments. No significant difference using unpaired t-test (**A-L**). C: classical and NC/I: non-classical/intermediate monocytes.
